# Curcumin (CUMINUP60®) mitigates exercise fatigue through regulating PI3K/Akt/AMPK/mTOR pathway in mice

**DOI:** 10.18632/aging.204614

**Published:** 2023-03-28

**Authors:** Minghui Hu, Muxuan Han, Hao Zhang, Zifa Li, Kaiyong Xu, Huaixing Kang, Jiancheng Zong, Feng Zhao, Yuanxiang Liu, Wei Liu

**Affiliations:** 1 Experimental Center, Shandong University of Traditional Chinese Medicine, Ji’nan, China; 2 Key Laboratory of Traditional Chinese Medicine Classical Theory, Ministry of Education, Shandong University of Traditional Chinese Medicine, Ji’nan, China; 3 College of Health Sciences, Shandong University of Traditional Chinese Medicine, Ji’nan, China; 4 Chenland Research Institute, Qingdao, China; 5 The First Clinical Medical College, Shandong University of Traditional Chinese Medicine, Ji’nan, China; 6 Department of Encephalopathy, The Second Affiliated Hospital of Shandong University of Traditional Chinese Medicine, Ji’nan, China

**Keywords:** curcumin, exercise fatigue, anti-fatigue effects, treadmill test, PI3K/Akt/AMPK/mTOR pathway

## Abstract

Curcumin is a chemical constituent extracted from *Curcuma longa L*. Several clinical and preclinical studies have demonstrated that it can mitigate exercise fatigue, but the exact mechanism is still unknown. Therefore, we applied a mouse model of exercise fatigue to investigate the possible molecular mechanisms of curcumin’s anti-fatigue effect. Depending on body mass, Kunming mice were randomly divided into control, caffeine (positive drug), and curcumin groups, and were given 28 days intragastric administration. Both the caffeine group and curcumin group showed significant improvement in exercise fatigue compared to the control group, as evidenced by the increase in time to exhaustion, as well as the higher quadriceps coefficient, muscle glycogen (MG) content, and increase in the expression of Akt, AMPK, PI3K, and mTOR proteins. While the curcumin group also significantly improved the exercise fatigue of the mice, demonstrating a lower AMP/ATP ratio and lactic acid (LA) content, and increased glycogen synthase (GS), and myonectin content compared to the caffeine group. Therefore, in the present study, we found that curcumin can exert a similar anti-fatigue effect to caffeine and may act by regulating energy metabolism through modulating the expression of the proteins in the PI3K/Akt/AMPK/mTOR pathway.

## INTRODUCTION

Exercise fatigue is a state of weakness, muscle pain, and reduced endurance after prolonged or strenuous exercise. This condition generally occurs during exercise and gradually increases as the body’s endurance is exhausted. It can last for days or even weeks after the exercise [1]. Short-lived fatigue is often thought to be a self-protective mechanism of the body obtained via evolution, which is extremely useful to maintain systemic homeostasis at all bodily levels, and can help us prevent intense exercise from causing a large depletion of energy stores and muscle damage [2, 3]. However, prolonged fatigue affects the body’s normal metabolism, leading to disturbances of the internal environment, and simultaneously, affecting the body’s mobility. Nowadays, many types of medications can be used to improve exercise performance, alleviate fatigue after intense exercise and promote organic recovery. Some antioxidant or anti-inflammatory substances, such as caffeine, vitamin E, vitamin C, Eicosapentaenoic Acid (EPA), and Docosahexaenoic Acid (DHA), have been reported to play more obvious roles in reducing exercise fatigue [4–6]. It seems that these drugs can be a decent treatment option to relieve post-exercise fatigue, however, the different disadvantages of these drugs, such as limited effects, toxicity, and low absorption and utilization rate [7, 8], have become a major impediment to their widespread use. Therefore, it is urgently necessary to find a medication that can relieve fatigue after exercise while having a high safety profile, low side effects, and rapid therapeutic effects.

Curcumin, a polyphenol derived from *Curcuma longa L*., has long been used as a traditional treatment for various diseases such as gastrointestinal, pulmonary, and liver problems [9–11]. The practices in the treatment of periodontitis, antiparasitic and anti-tumor therapy, showed that curcumin has achieved good therapeutic, indeed, with few side effects, and is easy to administrate [12–14]. In addition, modern pharmacology also proves that curcumin does have various biological activities [15, 16] and possesses the characteristics of weak toxic and side effects [17, 18], with a few research confirming its ability to mitigate exercise fatigue [19–21], but the mechanism behind this is still poorly understood. Because of the antioxidant and anti-inflammatory activity of curcumin, previous studies hypothesized that its anti-fatigue mechanism may be linked to glucose and lipid metabolism [10, 22] and tissue protection [23]. However, some clinical studies have confirmed that curcumin can significantly improve muscle vitality and recovery after strenuous exercise [24–26], we therefore speculated and tried to verify whether curcumin could improve muscle metabolism and thus take an anti-fatigue role in this manuscript.

## RESULTS

### Curcumin enhances the exercise performance of mice in treadmill tests and improves the physiological indices related to exercise fatigue

**Figure 1:**
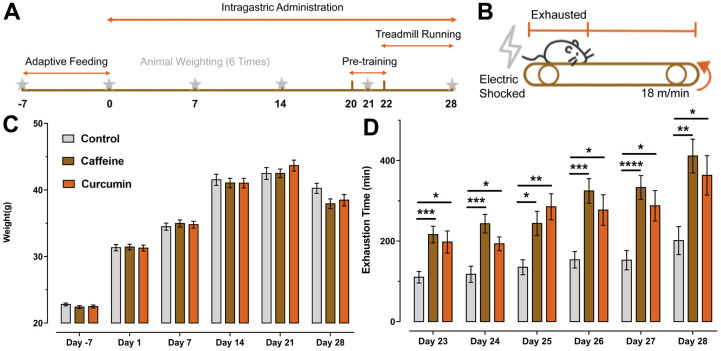
**Schedule of experiment design and the results of treadmill test and body weight.** (**A**) Arrangement of experimental design, after seven days of acclimatizing to the environment and weighing, the mice in the treatment group received 28 days of intragastric administration, during which they were weighed once a week. The treadmill test was carried out from d20 to d28, starting with a three-day pre-training period on d20, followed by a six-day formal experiment. (**B**) An illustration of the treadmill test, where the animal was moving at a speed of 18m/min on the track and was considered to have reached a state of exhaustion when it continued to move in the posterior third of the platform and received frequent electric shocks. (**C**) The results of body weight. On day -7(caffeine: p=0.432, curcumin: p=0.602), On day 1(caffeine: p=0.984, curcumin: p=0.996), On day 7(caffeine: p=0.804, curcumin: p=0.910), On day 14(caffeine: p=0.902, curcumin: p=0.892), On day 21(caffeine: p>0.999, curcumin: p=0.597), On day 28(caffeine: p=0.082, curcumin: p=0.277) compared to the control group. No significant differences could be observed in the time×column factor [F(10, 285) = 1.593, p=0.108] and the column factor [F(2, 57) = 0.237, p=0.790], but the time factor [F(2.290, 130.5) = 712.0, p<0.0001] did have a significant effect on the change in body weight of the mice. Data are shown as mean ± SEM. (**D**) Exhaustion time of the mice in the curcumin group and caffeine group (control group: n=22, dosed groups: n=19).

In the treadmill test, as the number of training days increased, the time to exhaustion increased in all groups of mice (Figure 1A, 1B), which demonstrated that treadmill training does improve the locomotor performance of mice and delayed the appearance of the physical fatigue [F (3.499, 199.4) = 22.38, *p*<0.0001]. We measured the weight of mice in each group every 7 days and found that there was no significant difference between the control group and the experiment group, so the interference of drug treatment on the constitution of mice was excluded (Figure 1A, 1C). After administration, mice in the caffeine and curcumin groups exhibited significantly longer time to exhaustion compared to mice in the control group (Figure 1D), revealing a significant effect of treatment interaction [F (2, 57) = 12.97, *p*<0.0001] on the exhaustion time. Although caffeine and curcumin treatment can cause a significant increase in exhaustion time compared with the control group, there is no significant difference between these two groups [F (1, 216) = 12.97, *p*=0.1375].

**Figure 2:**
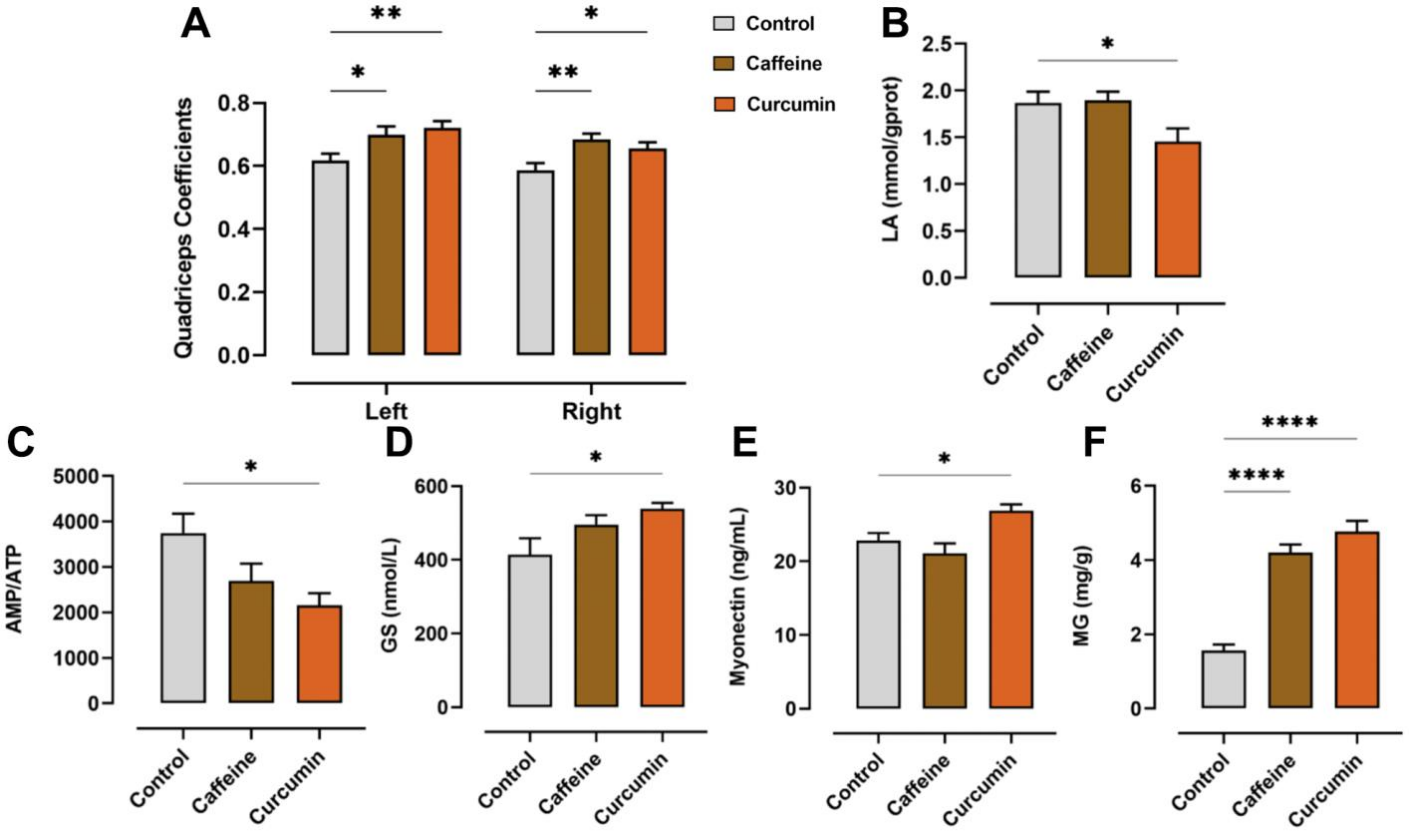
**The results of quadriceps coefficient and ELISA for AMP, ATP, LA, MG, GS, and myonectin.** (**A**) Quadriceps coefficient on both sides of the mouse (n=18). (**B**) LA content in the quadriceps muscle of mice. (**C**) The Ratio of AMP to ATP in the quadriceps muscle of mice (n=8, same below). (**D**) GS content of the quadriceps muscle in mice. (**E**) Myonectin content of the quadriceps muscle in mice. (**F**) MG content of the quadriceps muscle in mice. (*p<0.05, **p<0.01, ***p<0.001, ****p<0.0001 compared to the control group). Data are shown as mean ± SEM.

On the last day of the treadmill test, when the mice finished running, we removed the quadriceps muscles on both sides of the mice and then weighed them. The quadriceps coefficients were also calculated separately for each side by dividing the unilateral quadriceps weight by the body mass of the mice on the last day. We found an increase in quadriceps coefficients in both the caffeine (left: *p*=0.034, right: *p=*0.003) and curcumin (left: *p=*0.006, right: *p=0*.043) groups compared to the control group (Figure 2A). This suggested that after administration, the mice were able to develop a strong quadriceps muscle or keep the original quadriceps intact during the forceful exercise. A higher quadriceps coefficient also indicated that the muscle growth and endurance strength of the mice significantly increased with curcumin or caffeine treatment.

### Curcumin treatment could relieve fatigue by ameliorating energy metabolism

In our subsequent ELISA test, compared to the control group, the curcumin group exhibited a lower LA concentration (caffeine: *p=*0.979, curcumin: *p=*0.047) (Figure 2B), which was not seen in the caffeine group. Both dosing groups exhibited significantly higher MG content than the control group (*p<*0.0001 in both dosed groups) (Figure 2F). Moreover, the contents of GS (caffeine: *p=*0.146, curcumin: *p=*0.026) (Figure 2D) and myonectin (caffeine: *p=*0.448, curcumin: *p=*0.031) (Figure 2E) increased, which were not observed in the caffeine group also.

ATP is the basic energy regulator of cells and the direct energy supply material. Excessive body consumption of ATP during exercise is the main cause of fatigue. In this study, after the curcumin intervention, the ratio of AMP to ATP in muscle tissues was found to significantly decrease (caffeine: *p=*0.098, curcumin: *p=*0.012), indicating an increase in the relative content of ATP (Figure 2C).

### Curcumin regulates the proteins expression of PI3K/Akt/AMPK/mTOR pathway in mice with exhaustive exercise fatigue

**Figure 3:**
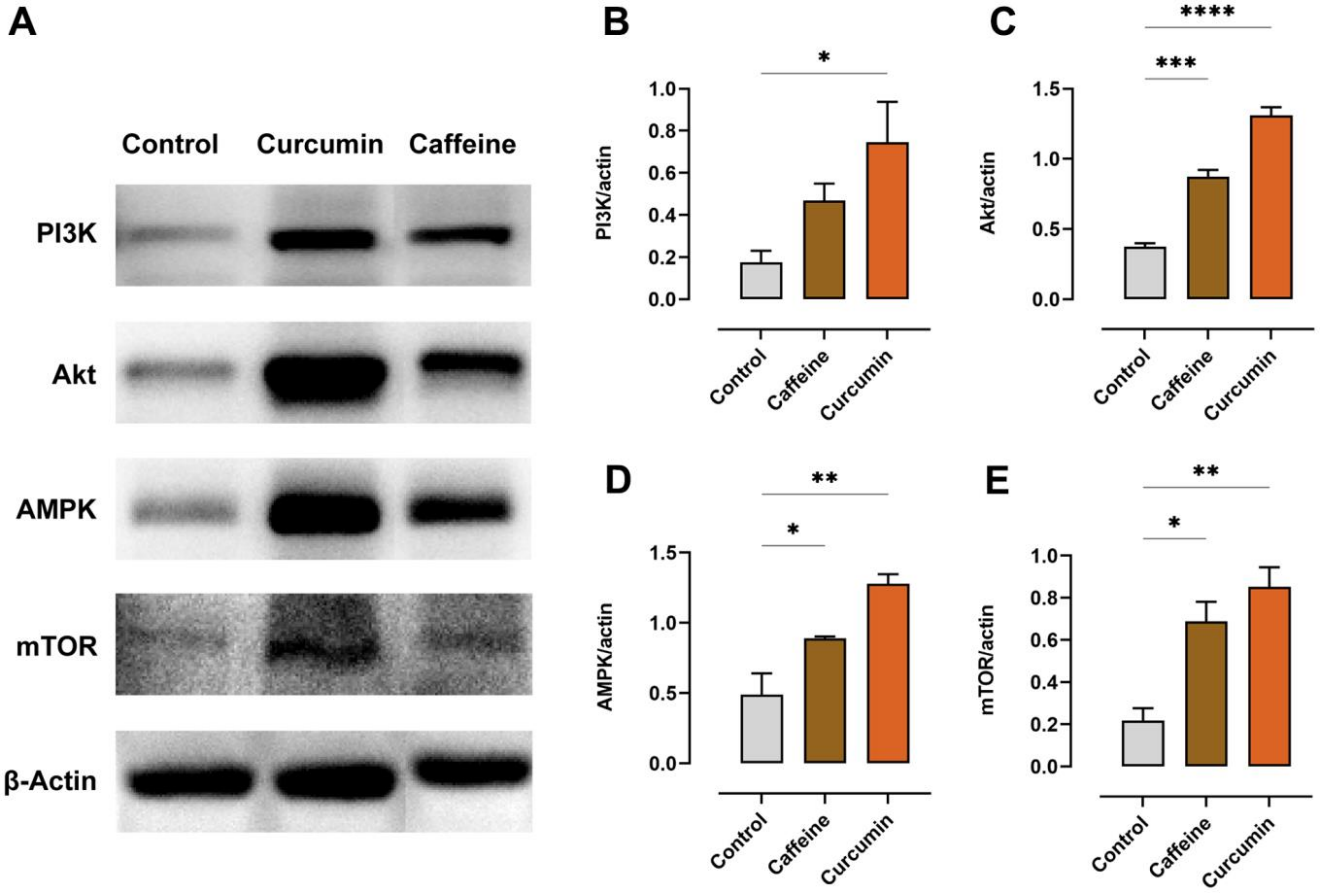
**Curcumin regulates the expression of the proteins in the PI3K/Akt/AMPK/mTOR pathway in the quadriceps of the exhausted mice (n=3).** (**A**) Protein expressions were detected via WB. The images were shown after stitching, the original images can be found in the Supplementary Material. (**B**) The protein content of PI3K. (**C**) The protein content of Akt. (**D**) The protein content of AMPK. (**E**) The protein content of mTOR. (*p<0.05, **p<0.01, ***p<0.001, ****p<0.0001 compared to the control group).

As a key physiological energy sensor, AMPK is the main regulator of energy balance between cells and organisms. PI3k/AKT can respond to changes in nutrients and metabolites, and mTOR is also a major regulator of cell growth and proliferation. Our WB results showed that after the exhaustive exercise, the exercise-fatigued mice produced altered expression levels of the proteins associated with the PI3K/Akt/AMPK/mTOR pathway (Figure 3A). The curcumin group showed increased expression of PI3K protein in the quadriceps (*p=*0.031) compared to the control group, while the caffeine group did not show significant differences (*p=*0.240) (Figure 3B). However, after administration, the Akt (caffeine: *p=*0.0004, curcumin: *p<*0.0001) (Figure 3C), AMPK (caffeine: *p=*0.044, curcumin: *p=*0.002) (Figure 3D), and mTOR (caffeine: *p=*0.012, curcumin: *p=*0.003) (Figure 3E) proteins were significantly increased in both dosed groups. Please note that as we have other bands in the WB test, so we have simply cut the bands to make it easier to present the results, all the original bands can be seen in the Supplementary Material (Supplementary Figure 1).

## DISCUSSION

**Figure 4:**
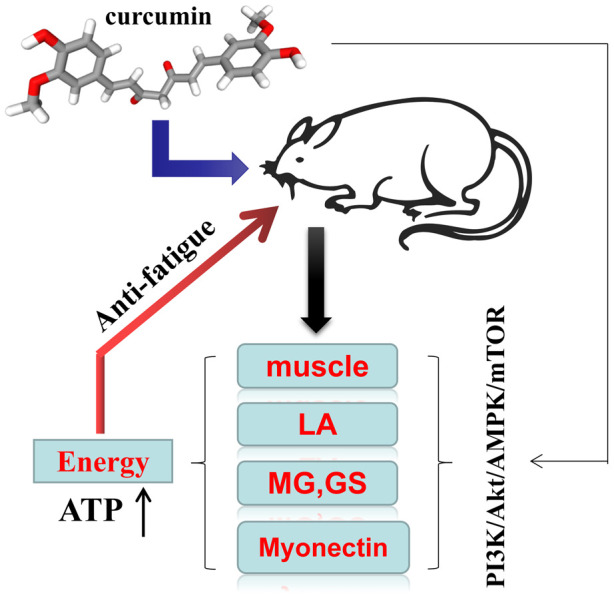
**Schematic diagram of the anti-fatigue mechanism of curcumin**. * Curcumin* intervention was shown to improve energy metabolism and reduce the accumulation of metabolic waste by regulating AMPK and PI3k/AKT-mTOR. LA: lactic acid, MG: muscle glycogen, GS: glycogen synthase.

In the present study, we examined the anti-effect of curcumin on exercise fatigue in mice. Increasing burnout time and muscle coefficient demonstrated that curcumin was effective in improving exercise tolerance and muscle strength in mice. A continuous intake of curcumin increased the MG, GS, and myonectin content in the quadriceps muscle of depleted mice while reducing the LA content and AMP/ATP ratio. At the same time, changes in these physiological markers indicate that curcumin, via intragastric administration, significantly reduced exercise fatigue in mice. Subsequent WB assays revealed that curcumin also upregulated protein expression of the PI3K, Akt, AMPK, and mTOR pathways in fatigue mice. We speculate that the curcumin supplement cut down fatigue and might improve energy metabolism upregulation of the PI3K/Akt/AMPK/mTOR pathway (Figure 4).

Previous studies have shown that when exercise fatigue occurs, the oxygen consumption of tissues will increase, and lead to the excessive consumption of antioxidants and synthetic antioxidant enzymes, which will weaken the ability to remove oxygen free radicals, and then damage the organelles of skeletal muscle cells, thus destroying the exercise ability [27, 28]. Curcumin has been reported could inhibit NF-κB and activate Nrf2 [29–31], which can eliminate oxygen free radicals by inhibiting oxidase, activating and protecting the antioxidant enzyme system, thus protecting the structure and function of the cell membrane and reducing cell damage [18, 32].

In recent years, some clinical studies have also shown that taking curcumin supplements has unexpected anti-fatigue effects. In a 12-week curcumin supplementation study, a significant decrease in muscle fatigue and muscle soreness scores was observed in the curcumin group [24], and another double-blind clinical research indicated that curcumin may facilitate faster recovery, slighter post-exercise pain, and reduced lactate accumulation and inflammatory factors content [25]. Interestingly, some studies have shown that although curcumin can alleviate exercise fatigue, some related inflammatory factors, such as TAC, TNF-α, or MDA, have not changed significantly [26]. Future research should have an in-depth investigation of the effect of curcumin supplementation on the mechanism of muscle recovery or anti-fatigue after exercise.

In our treadmill test, we did not observe significant differences between the body masses of the groups of mice (Supplementary Figure 1), which is consistent with the study of Wenching Huang [19]. Besides body masses, in our study, the mice in the administered groups showed differences in quadriceps coefficients bilaterally compared to the control group, which was not mentioned in Wenching Huang’s study [19]. A high muscle coefficient means that the proportion of exercise muscles increases, and the exercise ability and endurance increase accordingly, which may be one of the important reasons for the extension of fatigue time in the curcumin-treated group.

During the forceful treadmill exercise, the muscles require constant energy expenditure to maintain continuous movement of the body and produce a range of metabolites. The number of energy substances in the body, as well as the production of metabolites, can be our indicator to assess the degree of fatigue. For a short period at the beginning of exercise, ATP is the main energy supplier in the body, but as the exercise time increases, the supply of ATP cannot keep up with the rate of consumption of the organism, so the muscles begin to break down the stored glycogen in the body to obtain more energy, accompanied by the production of new glycogen. This resulted in a decrease in the AMP/ATP ratio [33, 34]. After the curcumin was administrated, the AMP/ATP ratio and the GS content significantly increased, and the glycogen consumption in the quadriceps muscle of mice was reduced, which is consistent with the findings of previous studies [19]. During this process, the lack of oxygen supply or hypoxia to the tissues due to strenuous exercise leads to anaerobic glycolytic metabolism of the muscles and tissues using glycogen and the production and accumulation of abundant LA. However, excessive muscle consumption of glycogen and accumulation of LA in the body can lead to fatigue and induce muscle or liver damage [35]. In addition, in the curcumin group, we identified increased levels of myonectin, a muscle factor that promotes fat intake and inhibits hepatic autophagy [36, 37]. Our experiments have demonstrated that curcumin can reduce the build-up of LA in the muscles and increase MG, GS, and myonectin levels to provide the body with sufficient energy to relieve fatigue, as well as protect the organism away from adverse factors.

Additionally, there are a lot of works suggesting that abnormal energy metabolism, which is associated with exercise fatigue, may be related to dysregulation of the PI3K/Akt/AMPK/mTOR signaling pathway [38–40], so we have mainly focused on investigating the changes in protein expression of key molecules in this pathway. The PI3K/Akt signaling pathway can promote myogenic cell proliferation and differentiation as well as the growth of the skeletal muscle [41]. In our experiments, the quadriceps coefficients of mice in the curcumin group increased significantly, which may be related not only to curcumin promoting the activation of the PI3K/Akt/mTOR pathway and thus the increase in GS activity [42] to increase the content of glycogen in muscle tissue but also to curcumin activating the PI3K/Akt signaling pathway causing muscle growth. In addition, previous research showed that curcumin can be used to treat metabolism-related diseases by regulating levels of various molecules, including Akt and AMPK [43]. Intracellular Akt protein phosphorylation could promote the activation of its downstream mTOR to promote protein synthesis [38], providing a certain material basis for cell growth. This is consistent with our results that the curcumin treatment group has significant muscle growth. And upregulation of mTOR can also cause an increase in myonectin, which is consistent with our findings [44]. The proliferation of skeletal muscle cells is essential for the recovery of muscles injured by exhaustive exercise, and curcumin can promote the proliferation of skeletal muscle cells through upregulation of the PI3K/Akt/mTOR pathway to achieve its anti-fatigue effect.

AMPK is thought to be critical in regulating energy metabolism in the body for it can sense the level of intracellular ATP. Under low energy supply conditions, such as during forceful exercise, phosphorylation of AMPK increases ATP production and decreases ATP consumption. When the cells need to use the stored energy, ATP is broken down into AMP, causing a decrease in the level of ATP, at which point the cell must reduce energy consumption to avoid ATP depletion. This is similar to our experimental results: the AMP/ATP ratio was reduced in the curcumin group and MG levels in the muscle were significantly increased compared to the control group, suggesting that curcumin may increase ATP production and reduce MG consumption by activating AMPK. Activation of the AMPK/mTOR signaling pathway also induces autophagy, and some research proved curcumin can reduce apoptosis under disadvantaged conditions by reducing oxidative stress and regulating cellular autophagy through the PI3K/Akt/mTOR and AMPK signaling pathways [36, 45]. In this case, the organism will be less dependent on external nutrients and will instead activate the mechanism of digesting its structural substances for energy, which will have a protective effect on the cells [46]. However, this is just speculation on our part at the moment and it still needs to be demonstrated by our further experimental exploration.

Caffeine has long been considered a classic ingredient to relieve various kinds of fatigue, so in our study, caffeine treatment was selected as a positive control group to comprehensively evaluate the anti-fatigue effect of curcumin. Caffeine, a methylxanthine compound, is the most popular central nervous stimulant in the world [17]. In nature, coffee beans, tea, and chocolate are all important sources of caffeine. Caffeine is a non-specific antagonist of adenosine A_1_ and A_2A_. Many studies have proved that caffeine has the effect of delaying fatigue in humans and experimental animals, and can significantly delay the occurrence of exercise fatigue [47, 48]. Its mechanism may involve the central and peripheral levels, but it may mainly act on the central [49]. Some studies have proven that caffeine may act on adenosine receptors and DA to reduce the impact of fatigue on motor ability and the central nervous system [50–52]. Other studies have shown that caffeine can increase fat decomposition and save glycogen to delay fatigue [53, 54], we also observed that the muscle glycogen level of mice in the caffeine treatment group was significantly higher than that in the control group (Figure 3F). It is noteworthy that our research found that caffeine treatment is consistent with curcumin treatment in many physiological indicators, although the degree of change is slightly different (Figures 3, 4). These results also show that anti-fatigue may be a problem of systemic metabolic changes in the body. To study this complex integrity issue, future research needs to use multi-channel omics to systematically obtain more comprehensive and accurate answers.

Of course, our experiment has some shortcomings, for example, we only discussed the effect of curcumin on exercise fatigue but did not investigate whether curcumin can relieve fatigue caused by other factors such as sleep disruption and lack of sleep. And only the mechanisms of signaling pathways related to energy metabolism were investigated, more mechanisms in other cases are yet to be designed to fully understand the anti-exercise fatigue effects of curcumin. We will improve these deficiencies in future experiments.

In summary, our study innovatively discusses the possible mechanisms and pathways of curcumin relieving exercise fatigue in mice, which has not been reported before, providing a reliable drug option for the relief of exercise fatigue. Based on the results of this experiment, we will further observe the changes in gene expression and transcript levels related to the PI3K/Akt/AMPK/mTOR pathway to better explain the neural mechanism of curcumin in relieving exercise fatigue, and we will also carry out experiments to identify the other signaling pathways related to exercise fatigue, to cross-sectionally investigate the effect of curcumin on pathways to refine the mechanism of curcumin’s ameliorative effect on exercise fatigue. We hope to provide a more comprehensive understanding of the mechanism of curcumin in relieving exercise fatigue by investigating from multiple perspectives and laying the foundation for its practical application.

## MATERIALS AND METHODS

### Animals

A total of 72 male SPF Kunming mice (18-22 g, 6-8 weeks old) were provided by Vital River Laboratories (Beijing, China) [Laboratory animal production license number: SCXK (Jing) 2016-0006]. After the adaptive feeding, the mice were randomly divided into 3 groups according to their body weight (the control group, the curcumin treatment group, and the caffeine treatment group). The entire experiment was carried out in a barrier environment under a temperature of 21±2° C and 12-h light/ dark cycles (lights on from 08:00 to 20:00 h) [Laboratory animal use license number: SYXK (Lu) 2017-0022]. The basal diet for each group was full nutritive feed pellets of an equal amount. Before the experiment, the animals were adapted to the new laboratory environment for 7 days. Body masses were recorded on days 0, 7, 14, 21, and 28 respectively (Supplementary Figure 1). The animal study was reviewed and approved by the Ethics Review Board of the Shandong University of Traditional Chinese Medicine [No. SDUTCM20210806007].

### Administration

Mice were intragastrically administrated at 9:00 am for 28 days (Figure 1A). Here, we used curcumin CUMINUP60^®^ (Chenland Nutritionals, Inc., powder, 58mg·kg^-1^, dissolved in purified water), which is obtained from the naturally extracted curcumin with excipients processed by micro-processing. The curcumin concentration of CUMINUP60^®^ is not less than 60% and it has improved the poor bioavailability of the original curcumin [23, 55, 56] by 8-20 times [52]. Besides the curcumin, we also added a positive drug caffeine (6mg·kg^-1^, dissolved in purified water too) as the potency comparison group [8]. Doses administered in the curcumin group were converted from the human equivalent dose (assuming a human weight of 60 kg, a mouse weight of 0.02 kg, and the dose of curcumin at 250 mg/day for humans) [57]. The control group was intragastric administration with the same volume of purified water.

### Treadmill test

We have made some changes to the existing treadmill test [58], the platform angle was removed and the electrical stimulation current was reduced to 1 mA to minimize the damage to the animal. Pre-training of the treadmill test was carried out three-day before the formal treadmill test (Figure 2A). 1 h after administration, all mice were first acclimatized for 15 min on the treadmill for three days. According to the result of the pre-training period, 18 m/min was the most suitable speed parameter that allowed the mice to perform the running task well and to exhaust within 5 h. The time to exhaustion was recorded and the criterion for exhaustion was that the mice fell off the grid continuously and get electrocuted, always in the posterior 1/3 of the runway, resting in ventral decubitus, breathing rapidly, and unable to keep running (Figure 1B). Animals were anesthetized with isoflurane 30 min after the end of the treadmill on the last day. The right and left quadriceps muscles were surgically removed and weighed, then quickly stored in liquid nitrogen for freezing.

### Enzyme-linked immunosorbent assay (ELISA)

The content of quadricep MG, LA, ATP (A043, A019, A095, Nanjing Jianceng Biology) and myonectin, GS, AMP (JL50241, JL13688, JL20649, Shanghai Jianglai Bio) were measured using ELISA kits. All procedures of the ELISA were conducted according to the kit manufacturer’s instructions. The optical density (OD) of each group was measured sequentially at 450 nm using a Microplate Reader (Multiskan SkyHigh, Thermo Scientific). The OD values of the samples are substituted into the regression equation, which is obtained from the standard curves made from the standards of the test kit, to find the concentration of each sample. As for the diluted test sample, the final sample concentration was multiplied by the dilution factor.

### WB analysis

The protein content of PI3K, Akt, AMPK, and mTOR in mouse quadriceps muscle was quantified using the Western-blot method. The tissues were rinsed with pre-chilled PBS (0.01M, pH=7.4) to remove residual blood and triturated with liquid nitrogen. The protein lysate (2×cocktail, 2% Triton-X100, 10mM Na_3_VO_4_, and 1mM PMSF dissolved in PBS) was added and cooled on ice for 10 min, followed by sonication. They were then centrifuged at 12000g at 4° C, and the supernatant was taken for WB. Equal amounts (50 μg) of total protein were separated by 10% SDS-PAGE gels (Sigma, USA) and transferred to PVDF membranes (Millipore, USA) and then blocked the membranes in 5% skim milk (containing TBS-T) at 25° C for 3 h. Then the corresponding primary antibodies; β-actin Mouse McAb (Wuhan SY, 66009-1-lg 1:5000), PI3K (ab191606, 1:1000, Abcam), Akt, AMPK, mTOR (CST 4691, CST 5831, CST 2972, 1:1000, Cell Signaling Technology) were added and incubation was performed overnight at 4° C. Then the samples were washed five times for 5 min each using TBST before incubating with the secondary antibodies (SA00001-1/2, 1:5000, Proteintech) at 25° C for 1 h. After a further 5 washes, place the membrane in the image capture apparatus, add ECL substrate (PE0010, Solarbio) under the light-proof conditions, and then capture the image. The gray-scale value of each group of target protein bands was analyzed using Image J software.

### Statistical analysis

Statistical analyses were performed using GraphPad Prism 8 (GraphPad Software, La Jolla, CA, United States). All data meet the normal distribution and have equal variance. Then the two-way analysis of variance (ANOVA) multiple comparisons and Tukey’s test were carried out for the exhaustion time, while other indicators were analyzed using one-way ANOVA followed by Dunn’s test. Data were presented as the mean ± standard error of the mean (SEM) and *p*-values less than 0.05 were considered statistically significant.

## Supplementary Material

Supplementary Figure 1
