# Supplementary material for: Curcumin (CUMINUP60®) mitigates exercise fatigue through regulating PI3K/Akt/AMPK/mTOR pathway in mice
Source: Aging (Albany NY). 2023 Mar 28;15(6):2308–20. doi: 10.18632/aging.204614 (PMC10085593; doi:10.18632/aging.204614)
Supplement: Supplementary Figure 1 [file aging-15-204614-s001.pdf]

SUPPLEMENTARY FIGURE

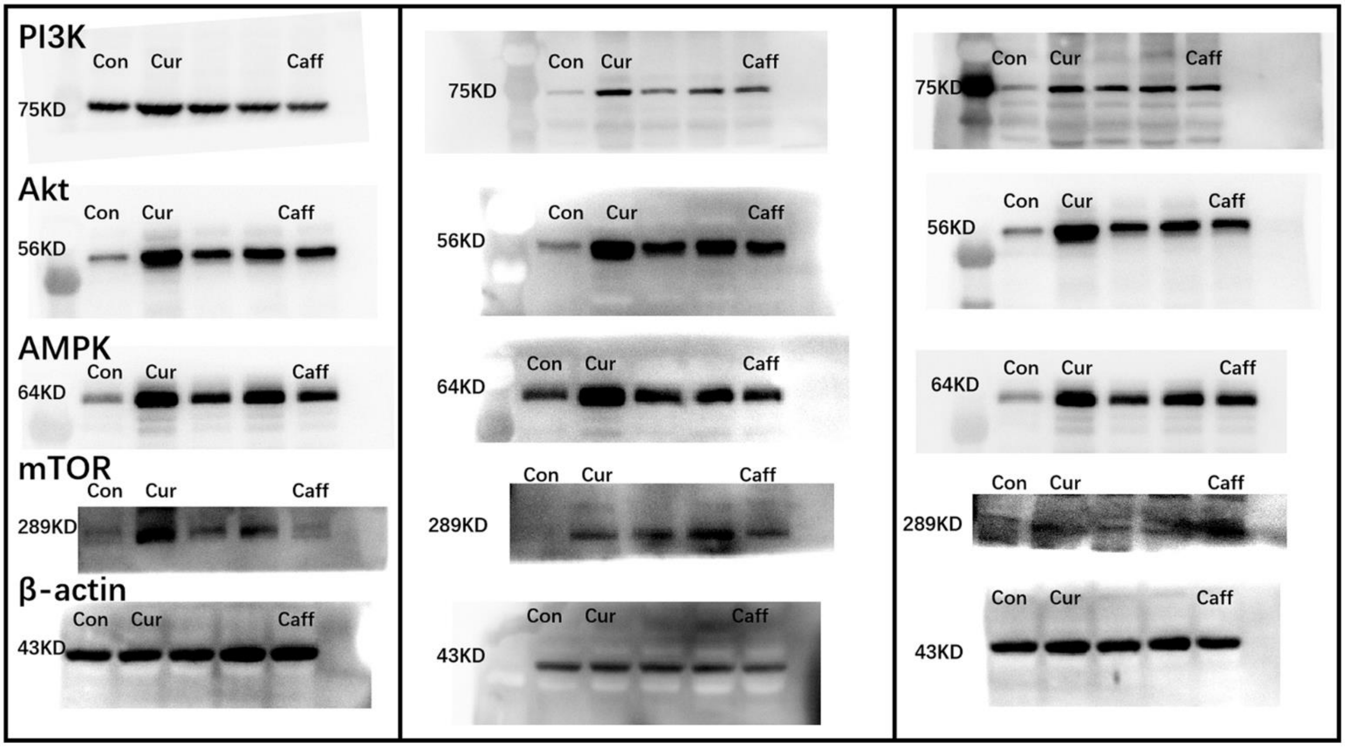

Supplementary Figure 1. The original images of the expression of the proteins in PI3K/Akt/AMPK/mTOR were detected via WB for three repeats. Columns 1, 2, and 5 are the control, curcumin, and caffeine groups, respectively. To present the results more visually, we have removed the two unrelated bands between the curcumin and caffeine groups in the paper.
